# Fine-tuning the regulation of Cas9 expression levels for efficient CRISPR-Cas9 mediated recombination in *Streptomyces*

**DOI:** 10.1007/s10295-020-02277-5

**Published:** 2020-05-04

**Authors:** Suhui Ye, Behnam Enghiad, Huimin Zhao, Eriko Takano

**Affiliations:** 1 Department of Chemistry, School of Natural Sciences, Faculty of Science and Engineering, Manchester Centre for Synthetic Biology of Fine and Speciality Chemicals (SYNBIOCHEM), Manchester Institute of Biotechnology, University of Manchester, 131 Princess Street, Manchester, M1 7DN UK; 2 Present Address: Research Group BIONUC (Biotechnology of Nutraceuticals and Bioactive Compounds), Departamento de Biología Funcional, Área de Microbiología, IUOPA (Instituto Universitario de Oncología del Principado de Asturias), ISPA (Instituto de Investigación Sanitaria del Principado de Asturias), Universidad de Oviedo, Avenida Julián Clavería S/N, 33006 Oviedo, Principality of Asturias Spain; 3 Department of Chemical and Biomolecular Engineering, and Carl R. Woese Institute for Genomic Biology, University of Illinois At Urbana-Champaign, Urbana, IL 61801 USA

**Keywords:** *Streptomyces*, CRISPR-Cas9, Theophylline riboswitch, Genome editing, Glycerol

## Abstract

CRISPR-Cas9 has proven as a very powerful gene editing tool for *Actinomyces*, allowing scarless and precise genome editing in selected strains of these biotechnologically relevant microorganisms. However, its general application in actinomycetes has been limited due to its inefficacy when applying the system in an untested strain. Here, we provide evidence of how Cas9 levels are toxic for the model actinomycetes *Streptomyces coelicolor* M145 and *Streptomyces lividans* TK24, which show delayed or absence of growth. We overcame this toxicity by lowering Cas9 levels and have generated a set of plasmids in which Cas9 expression is either controlled by theophylline-inducible or constitutive promoters. We validated the targeting of these CRISPR-Cas9 system using the glycerol uptake operon and the actinorhodin biosynthesis gene cluster. Our results highlight the importance of adjusting Cas9 expression levels specifically in strains to gain optimum and efficient gene editing in *Actinomyces*.

**Electronic supplementary material:**

The online version of this article (10.1007/s10295-020-02277-5) contains supplementary material, which is available to authorized users.

## Introduction

Since its first biotechnological application, CRISPR-Cas-based technology has marked a turning point in synthetic biology [1, 2]. Thus, the diversity in applications and the range of organisms genetically modified using CRISPR-Cas9 system have increased exponentially during the past few years ([3, 4] for detailed reviews). However, this breakthrough technology is still in its infancy, especially in actinobacteria, with numerous proof-of-concept studies supporting its wide applicability but hindering its routine use, where it is often unsuccessful.

Actinobacteria, with *Streptomyces* as its major representative, are G-C rich Gram-positive bacteria renowned for their natural ability to produce a plethora of pharmacologically active and industrially relevant natural products. In fact, they are known to produce over two-thirds of all known secondary metabolites [5]. To combat antimicrobial resistance it is pertinent to elucidate novel bioactive compounds. The current search for new natural products is focused on the awakening of sleeping biosynthesis gene clusters that are found through genome sequences of many microbes [6]. To awaken these potential biosynthesis gene clusters, it will be much faster and efficient to be able to genetically engineer the genome. However, genetic engineering of these bacteria remains challenging. CRISPR-Cas9 has emerged as a very promising alternative [7]. The endonuclease Cas9, guided by a customer-designed short RNA guide transcript, generates a double strand break (DSB) in a specific point of the DNA target. This, combined with homology-directed repair (HDR) system in *Streptomyces*, allows rapid, precise and efficient scarless genetic edition [4].

The four most widely used CRISPR-Cas9 toolkits to perform in vivo genome editing in *Streptomyces* are pCRISPomyces-2 [8], pKCcas9dO [9], pCRISPR-Cas9 [10] and pWHU2653 [11] (for a detailed and critical review of these toolkits features and their successful application to a wide range of actinomycetes, see Alberti and Corre [12]). However, these and other authors reported a series of crucial drawbacks in its application in *Streptomyces*, ranging from the absolute absence of exconjugants [13] to the opposite situation with zero genome-edited colonies among all exconjugants obtained [14]. There are several factors that might affect CRISPR-Cas9 system efficacy, for some of which specific solutions have been adopted. (1) The pSG5 origin of replication, present in most of CRISPR-Cas9 bearing plasmids, can promote integration of the plasmid into the chromosome when repeated regions are targeted. This problem can be solved by using the defective pIJ101 origin of replication instead [15]. (2) The guide RNA selection plays an important role in the outcome of the recombination event, as different guide RNAs targeting the same gene can lead to differences in Cas9 cleaving efficiency [10]. This issue can be addressed by testing several protospacers for each target [9]. (3) The DSBs caused by Cas9 can lead to genome instability, a problem that has recently been addressed by using a nickase version of Cas9 fused to a cytidine or adenosine deaminase-based editor [16]. (4) Cas9 expression may be toxic for the bacteria, as previously suggested by other authors [8, 16, 17]. Recently, a multi-layer control of Cas9 activity coupled with ATP-synthase over expression has been successfully adopted by Wang and colleagues, to increase transformation efficiency in *S. coelicolor*, showing different levels of genome editing efficiency depending on the condition [17]. In their study, Cas9 levels are controlled at transcription, translation and protein level, by using thiostrepton, theophylline and blue light as respective inductors. Only by addition of the three inducers, the authors were able to achieve values higher than 10% editing efficiency.

In this work, we investigated the effect of Cas9 expression on cell viability in the model actinomycete *S. coelicolor* M145 and the related *S. lividans* TK24, confirming Wang and colleagues’ previous observations. Unlike them, we performed a set of experiments that clearly and rapidly showed the range of tolerance to Cas9 levels for each strain, in regard to both survival and Cas9 activity. We also produced a new plasmid toolkit to perform CRISPR-Cas9 mediated recombination in which Cas9 expression is either tightly translationally regulated by theophylline or controlled by a relatively weak promoter for both strains. The advantages of using each of the proposed systems are further discussed. All experiments conducted showed clear differences between the level of inductor required by each strain, emphasizing the importance of adjusting Cas9 levels to each strain to achieve the optimum balance between survival and editing efficiency. We believe that this study highlights the importance of pre-exploring the tolerance to Cas9 expression when this technology is intended to be applied in a new specie and provides with tools that can be accordingly adapted for genome editing the targeted strain.

## Methods

### Bacterial strains and culture conditions.

*Streptomyces coelicolor A3*(*2*)*,* M145 [18] and *Streptomyces lividans* TK24 [19] were used as source of DNA and for conjugation experiments. *Streptomyces* strains were grown on solid soy flour mannitol (SFM) [18], liquid tryptic soy broth (TSB) (Oxoid) and MYG medium [8] for conjugation efficiency and CRISPR-Cas9 induction experiments; and on solid minimal medium (SMM) [18] prepared with glucose or glycerol as sole carbon source for the screening of positive recombinants targeting the glycerol uptake operon. A 40-mM stock of theophylline-anhydrous (Sigma Aldrich) was prepared in distilled water and, after filter-sterilisation, added to the media at concentrations between 0.5–12 mM. *E. coli* DH5α (NEB) was used for routine cloning, and *E. coli* ET12567[pUZ8002] [19, 20] was used as donor in the intergeneric conjugation from *E. coli* to *Streptomyces*. *E. coli* strains were grown in Luria–Bertani (LB) broth (Formedium). All bacterial strains used in this work are listed in Table 1. When required antibiotics were added to the media as follows: apramycin (50 μg ml^−1^), chloramphenicol (25 μg ml^−1^), kanamycin (50 μg ml^−1^) and nalidixic acid (25 μg ml^−1^) for all media condition and for *Streptomyces* and *E. coli*.

**Table 1:** Strains and plasmids used in this study

Strain or plasmid	Genotype/characteristics	Use	Reference(s) or source
Strains			
*Escherichia coli* DH5α	*fhuA2 Δ(argF-lacZ)U169 phoA glnV44 ϕ80Δ(lacZ)M15 gyrA96 recA1 relA1 endA1 thi-1 hsdR17*	Cloning	NEB
*Escherichia coli* ET12567[pUZ8002]	*dam13::Tn9 dcm6 hsdM hsdR recF143 zjj201::Tn10 galK2 galT22 ara14 lacY1 xyl5 leuB6 thi1 tonA31 rpsL136 hisG4 tsx78 mtli glnV44 F − ,* carries plasmid pUZ8002	Intergenic conjugation	[18, 19]
*Streptomyces coelicolor* M145	Wild type		[17]
*Streptomyces coelicolor A3(2)*	Wild type		[17]
*Streptomyces lividans* TK24	Wild type		[17]
Plasmids			
pCRISPomyces-2	*oriT, rpsLp (XC)-cas9*		[8]
pSET152	*oriT-traJ*	Source of *oriT-traJ*	[24]
pCRISPomyces-ptc	*oriT, ermEp1-TheoR-cas9*	Source of *TheoR*	A. Rodríguez-García (unpublished)
pCM4.4	oriT-traJ, ermE*p-cas9		This work
pCM4.4-Act-KO	oriT-traJ, ermE*p-cas9, spacer sequence, DNA repair template	CRISPR-Cas9-mediated deletion of actinorhodin positive transcriptional regulator	This work
pCM2.1	*oriT-traJ, rpsLp (XC)-cas9*	Test effect of *traJ* lack	This work
pCMU	*oriT-traJ, rpsLp(XC)-TheoR-cas9*	Test effect of Cas9 levels	This work
pCMU-4	*oriT-traJ, D4-TheoR-cas9*	Test effect of Cas9 levels	This work
pCMUtuf	pCMU, *tufprot*	Control of Cas9 activity	This work
pCMU-4tuf	pCMU-4, *tufprot*	Control of Cas9 activity	This work
pCM(-cas9)	pCMU-4, *Δcas9*	Control of Cas9 expression	This work
pCMU-4dGly	pCMU-4, *glyprot*, DNA repair template	CRISPR-Cas9-mediated interruption of glycerol uptake operon	This work

### DNA manipulation and vectors

DNA manipulations were performed according to standard procedures for *E. coli* [21] and *Streptomyces* [18]. Fragment amplifications for plasmid construction were carried out with PrimeSTAR Max DNA polymerase (Takara) whilst amplifications from spores were performed with Terra polymerase (Takara). Constructs were assembled by Gibson Isothermal Assembly with NEBuilder HiFi DNA Assembly Master Mix (New England Biolabs, NEB), following the manufacturer’s instructions. When plasmid DNA was used as template for fragment amplification, a *Dpn*I restriction digest was performed after PCR in order to remove all methylated (template) DNA. When indicated, unique nucleotide sequences (UNS) were added as overlapping regions to increase the efficiency of the Gibson assembly [22]. The correct assembly was assessed by restriction digest and Sanger sequencing (Eurofins Genomics). The gBlocks fragments were ordered from Integrated DNA Technologies (IDT).

### Plasmid construction

pCM2.1 is a derivative of pCRISPomyces-2 [8] in which *traJ* was cloned between *oriT* and *rpsLp (XC)*. It was constructed by 2-fragment Gibson assembly: a 6,665-bp fragment containing *gapdhp-lacZ-gRNA-tracr-ColE1ori-aac3(IV)-pSG5rep-oriT-traJ* and a 4,476-bp fragment containing *rpsLp(XC)-cas9*. The former was amplified from pCM4.4 (this manuscript) using primers oriT rev Gibson and pCM fw Gibson; whilst the latter was amplified from pCRISPomyces-2 with primers rpsLp fw Gibson and Cas9 rev Gibson.

pCMU is a derivative of pCM2.1 in which the theophylline riboswitch (*TheoR*) was cloned between *rpsLp(XC)* and the ribosome binding site (RBS) of *cas9*. It was constructed by 2-fragment Gibson assembly: a 6,939-bp fragment containing *gapdhp-lacZ-gRNA-tracr-ColE1ori-aac3(IV)-pSG5rep-oriT-traJ-rpsLp(XC)* and a 4,535-bp fragment containing *TheoR-cas9*. The former was amplified from pCM2.1 using primers rpsLp rev Gibson and pCM fw Gibson; whilst the latter was amplified from pCRISPomyces-ptc (A. Rodríguez-García, unpublished) with primers Cas9 fw Gibson and Cas9 rev Gibson.

pCMU-4 is a derivative of pCMU in which the *rpsLp(XC)* was replaced by *D4* promoter [23]. For that purpose, *D4* sequence was inserted de novo as an overlapping region by including its sequence to the corresponding primers. It was constructed by 2-fragment Gibson assembly: a 6686-bp fragment containing *gapdhp-lacZ-gRNA-tracr-ColE1ori-aac3(IV)-pSG5rep-oriT-traJ-D4* and a 4280-bp fragment containing *D4*-*TheoR-cas9*. Both fragments were amplified from pCMU, the former using primers pCM fw Gibson and pCMU-4 Gibson rev, whilst the latter used primers pCMU-4 Gibson fw and Cas9 rev Gibson.

pCMUtuf and pCMU-4tuf were used as controls of Cas9 activity. CRISPy-web [24] was used to obtain a list of protospacers within *tuf1* coding sequence, and 2 protospacers cutting at both ends of the gene were selected. For that purpose, a synthetic dsDNA gBlocks containing 2 protospacers targeting *S. coelicolor tuf1* (SCO4662) was designed with the configuration of (*Bbsa*I site-*protospacer1*-*tracRNA*-*T7terminator*-*gapdhp*-*protospacer2*-*Bbsa*I site) (Table S1) and sourced to IDT. The *tuf1prot* synthetic DNA was cloned into pCMU and pCMU-4tuf by a *Bbs*I-mediated Golden Gate assembly to give rise to pCMUtuf and pCMU-4tuf, respectively. Correct insertion was verified by Sanger sequencing with primer Fragment seq rev.

pCM(-cas9) is a derivative of pCMU-4 in which the *cas9* coding region has been removed. For that purpose, the 6,686-bp fragment used to construct pCMU-4 was 5′-phosphorylated with T4 PNK (NEB) and subsequently re-ligated with T4 DNA ligase (NEB).

pCMU-4dGly is a derivative of pCMU-4 designed to disrupt the glycerol uptake operon by CRISPR-Cas9. Both protospacer and homology arms were assembled in one step via 5-fragment Gibson assembly. To facilitate the efficiency of the reaction, UNS sequences were added to every overlapping region except the one with the protospacer. Thus, a 2,305-bp comprising *protospacer-gRNA-tracr-aac3(IV)-UNS1* was amplified with primers dgly GA Prot1 fw and dgly GA UNS1 rev. A 1,312-bp fragment containing *UNS1-5′ homologous arm-UNS3* was amplified with primers dgly F1 UNS1 fw and dgly F1 UNS3 rev, whereas *UNS3-3′ homologous arm-UNS9* was amplified as a 1,222-bp fragment with primers dgly F2.1 UNS3 fw and dgly F2 UNS9 rev. A 2,501-bp fragment comprising *UNS9-pSG5rep-UNS2* was amplified with primers dgly GA UNS9 fw and dgly GA UNS2 rev. Finally, a 5442-bp fragment containing *UNS2-oriT-traJ-D4-TheoR-cas9-gapdhp-protospacer* was amplified with primers dgly GA UNS2 fw and dgly GA Prot1 rev. All fragments were amplified from pCMU-4, with the exception of the ones corresponding to the homology arms, for which *S. coelicolor* M145 genomic DNA was used as template.

pCM4.4 is a derivative of pCRISPomyces-2 in which Cas9 promoter *rpsLp(XC)* was replaced with ermE* promoter and a new RBS sequence, and full *traJ* sequence was cloned between *oriT* and ermE*. First, a 461-bp dsDNA synthesized gBlocks fragment containing ter-ermE*p-RBS, and a section of *cas9* gene was cloned into a 10,453-bp fragment created by digestion of pCRISPomyces-2 by restriction enzyme *Sbf*I using 2-fragment Gibson assembly. The full *traJ* and *oriT* sequence was then amplified from plasmid pSET152 [25] by PCR and cloned into the previously constructed vector digested by restriction enzymes *Avr*II and *Nhe*I using 2-fragment Gibson assembly to yield the final pCM4.4 construct.

pCM4.4-Act-KO is a derivative of pCM4.4 designed to knock out the positive pathway regulator for actinorhodin biosynthetic gene cluster. The gRNA spacer sequence was assembled into the plasmid as described before [8]. The 1.5-kB homology arms were amplified from *S. coelicolor A3(2)* genomic DNA by PCR. pCM4.4 containing gRNA sequence was linearized by *Xba*I and the homology arms were assembled into the plasmid using 3-fragment Gibson assembly.

All plasmids used in this work are summarized in Table 1 and the corresponding map can be found in supplementary Fig. S1. Primers are listed in Table S1.

### Intergeneric conjugation and CRISPR-Cas9 system induction

Plasmids were delivered into *Streptomyces* by intergeneric conjugation according to standard procedures [19]. An overnight culture of *E. coli* ET12567[pUZ8002] containing the corresponding plasmid was diluted 1:100 into fresh LB with antibiotics and grown to an OD_600_ of 0.6. Then, 1 mL of the culture was washed twice and resuspended in the same volume of fresh LB without antibiotics. The washed cells were mixed with 10^8^ of pre-germinated spores in a 5:1 volume ratio. 100 μL from the mixture was spread on SFM supplemented with 0, 0.5, 1 or 2 mM of theophylline. Plates were incubated at 30 °C for 16 h, subsequently overlaid with 1 mL of distilled water containing nalidixic acid (25 μg ml^−1^) and apramycin (50 μg ml^−1^) and incubated at 30 °C. All plates were photographed 6 days after conjugation and the number of exconjugants counted. The conjugation efficiency was calculated per conjugation by multiplying the number of exconjugants per plate by the dilution factor.

For the glycerol uptake operon disrupted by CRISPR-Cas9 mediated recombination, 5 exconjugants from the uninduced conjugation plate (0 mM of theophylline) were pooled together in 400 μL of TSB, thoroughly mixed by vortexing and 100 μL of the mixture was used to inoculate 10 mL of TSB containing apramycin (50 μg ml^−1^), nalidixic acid (25 μg ml^−1^) and 4, 8 or 12 mM of theophylline. Cultures were incubated at 30 °C and 220 rpm for 4 days and afterwards re-streaked on SFM for single colonies. Ten colonies from each condition were randomly picked, subcultured twice for single colonies on SFM and subjected to further screening.

### Screening of mutants

To determine the Cas9 editing of glycerol uptake operon, screening was conducted by both phenotype and DNA verification by analysing ten independent mutants. For each screened colony, half of the colony was picked and plated out on SMM containing either glycerol or glucose. Plates were incubated at 30 °C and photographed after 6 days. The other half of the colony was subjected to PCR directly from spores with two pairs of primers. The set of primers ‘dGly’ (‘dGly SCO check fw’ and ‘dGly SCO check rev’) can amplify a region containing the deleted sequence, exclusively from the chromosome, as ‘dGly SCO check fw’ anneals outside of the fragment used as the 5′ homologous arm for the recombination (Fig. S2). Thus, a 1360-bp fragment was expected from the mutant strain in contrast to the 3032-bp fragment expected from the wild type. A second pair of primers, ‘SCO1660′ (‘SCO1660 check fw’ and ‘SCO1660 check rev’), amplified a 775-bp fragment within the deleted region. Therefore, no amplification was expected from the mutant strain (Fig. S2).

To determine the Cas9 editing of actinorhodin positive regulator gene, *act II*-ORF4, eight exconjugants were picked and streaked on SFM plates supplemented with nalidixic acid (25 μg mL^−1^). The plates were incubated at 37 °C so that the editing plasmid is lost. After 3 days of incubation, spores from each exconjugant were picked and transferred into 3 mL liquid MYG medium. The cells were incubated at 37 °C for 2 days and their genomic DNA was extracted using Wizard genomic DNA extraction kit (Promega) following the manufacturer’s protocol. 50 ng of genomic DNA was used as template for genotyping PCR. For positive PCR primer pair of ‘Act-edit-F’, and ‘Act-edit-R’ were used. For negative controls, primer pairs (‘Act-edit-F’, ‘Act-WT-R’) and (‘Act-edit-R’, ‘Act-WT-F’) were used.

## Results and discussions

### Effect of Cas9 expression on conjugation efficiency

Our previous attempts to deliver plasmid pCRISPomyces-2 [8] into either *Streptomyces coelicolor* M145 or *Streptomyces lividans* TK24 through intergeneric conjugation appeared to be unsuccessful. There are published reports from other authors of failed attempts to obtain exconjugants with the same plasmid in *S. coelicolor* [13], *Streptomyces* sp. KY 40–1 [26], *Streptomyces* sp. NRRL S-244 and *S. roseosporus* NRRL 15,998 [27]. All these together suggested an existing issue with the plasmid that prevented any exconjugant from growing.

**Fig. 1:**
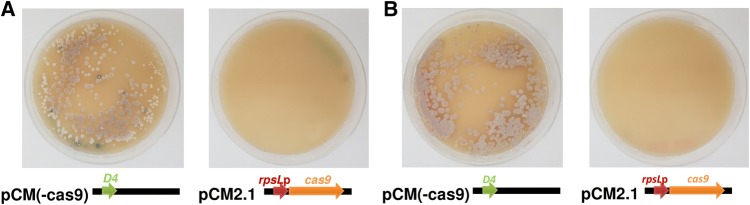
Plates incubated for 6 days after conjugation with pCM2.1 (pCRISPomyces-2 derivative with *traJ* amended and *rpsL*p promoter in front of *cas9*) and pCM(-cas9) (control plasmid with D4 promoter but lacking *cas9*) in **a***S. coelicolor* and **b***S. lividans*

An analysis of pCRISPomyces-2 nucleotide sequence revealed that *traJ* gene coding sequence in this plasmid is incomplete, encoding a truncated and likely non-functional protein. TraJ positively regulates the expression of transfer genes that are involved in the conjugal transfer of DNA between bacterial cells [28]. Despite not being essential for the process, previous observations indicated that its absence in the conjugable plasmid greatly affects the efficiency of the conjugation process [29]. Therefore, *traJ* sequence was amended to encode for a complete TraJ in pCRISPomyces-2 to create plasmid pCM2.1. However, as was the case with the parental pCRISPomyces-2 plasmid, no exconjugants grew after conjugations with both *S. coelicolor* and *S. lividans*, ruling out the absence of functional TraJ as the reason for the lack of exconjugants. Multiple exconjugants were observed from conjugation with only pCM(-cas9) plasmid, a pCMU-4 derivative lacking *cas9*, suggesting that the problem of not obtaining the exconjugants may be due to Cas9 expression (Fig. 1).

In pCRISPomyces-2, *cas9* expression is under the control of *rpsLp (XC)*, the promoter from 30 s ribosomal protein S12 of *Xylanimonas cellulosilytica*, which had been proved to be the strongest promoter among a collection of natural promoters from *Streptomyces griseus* and other Actinobacteria, tested in *S. lividans* [30]. In fact, it showed more than 100-fold increased activity compared to the commonly used ermE*p. In order to test if *rpsLp (XC)* is driving the expression levels of *cas9* so high that it might become toxic for the cell, plasmid pCMU was built by cloning the theophylline riboswitch [31] between *rpsLp (XC)* and the RBS of *cas9*. This enabled the *cas9* expression to be inducible by theophylline. Plasmid pCMUtuf, containing two protospacers targeting *tuf1* but no homology repair template, was used as a control to analyse the Cas9 activity. This gene encodes the elongation factor Tu (EF-Tu), which is an essential protein involved in protein translation [32]. A conjugation in parallel with pCM(-cas9) was performed as a positive control of the conjugation process.

When pCMU was delivered into *S. coelicolor*, exconjugants were observed in all conditions, indicating that Cas9 levels had been reduced to non-toxic levels for the cell. A negative correlation between the number of exconjugants and the concentration of theophylline is observed: 13,200 exconjugants in the uninduced condition, 8208 at 0.5 mM, 7044 at 1 mM and 3780 at 2 mM of theophylline. Considering the number of exconjugants observed in the uninduced condition as 100% survival, induction with 0.5 mM of theophylline reduced survival to 62%, 1 mM to 53% and 2 mM to 28% (Table 2, Fig. S3a). The same trend was also observed with pCM(-cas9): 10,140 (100%) in the uninduced condition, 5640 (55%) at 0.5 mM, 6000 (59%) at 1 mM and 1800 (17%) at 2 mM of theophylline (Table 2, Fig. S3a); suggesting that that negative effect observed with pCMU and pCM(-cas9) may be mainly caused by the toxicity of theophylline to *S. coelicolor* rather than Cas9, which has not been reported previously. When Cas9 activity was tested with pCMUtuf, no exconjugants were observed in any of the conditions assayed, included the uninduced condition. This suggests that the inducible theophylline riboswitch system is leaky and basal activity of Cas9 is high enough to be lethal for the cells.

**Table 2:** Number of exconjugants^a^ and percentage of survival^b^ from Cas9 tolerance experiments

Plasmid	Theophylline concentration (mM)			
	0	0.5	1	2
*Streptomyces coelicolor* M145				
pCM2.1	0	N/A	N/A	N/A
pCM(-cas9)	10,140^a^/100^b^	5,640/55	6,000/59	1,800/17
pCMU	13,200/100	8,208/62	7,044/53	3,780/28
pCMUtuf	0	0	0	0
pCMU-4	11,760/100	8,124/69	5,712/49	1,440/12.2
pCMU-4tuf	10,560/100	36/0.003	0	0
*Streptomyces lividans* TK24				
pCM2.1	0	N/A	N/A	N/A
pCM(-cas9)	19,320/100	16,920/88	12,960/67	3,276/17
pCMU	0	0	0	0
pCMUtuf	0	0	0	0
pCMU-4	18,000/100	12,516/70	9,516/53	1,560/9
pCMU-4tuf	20,304/100	5,760/28	3,480/17	240/0.01

^a^Number of exconjugants expressed as number of colony-forming units (CFUs) per conjugation

^b^Percentage of survival considering number of CFUs in the uninduced condition (0 mM theophylline) as 100%

In case of *S. lividans,* no exconjugants were observed 6 days after conjugation with pCMU, including the uninduced condition (Table 2, Fig. S3b). This suggests that in this organism, and unlike in *S. coelicolor*, basal expression of Cas9 is toxic at this level. However, small colonies started to appear when plates were incubated for 10 days (Fig. S5). This indicates that for *S. lividans* toxicity leads to delayed growth instead of lethality. Even though *S. lividans* can eventually overcome Cas9 negative effects, and, therefore, plasmids like pCRISPomyces-2 can be successfully used for genome editing as previously reported [8, 33], it requires a very long time. It would be better if editing system can be completed in a shorter timeframe.

### Lowering Cas9 levels to obtain a tightly regulated Cas9 expression system.

In light of the results from pCMU, it became clear that Cas9 expression needed to be further lowered. Therefore, plasmid pCMU-4 was created, in which *rpsLp (XC)* promoter was replaced by *D4* promoter [24]. *D4* promoter was shown as the weakest promoter among all 200 natural and synthetic promoters reviewed by Myronovskyi and Luzhetskyy [34]. As with pCMU, control conjugations in parallel with pCMU-4tuf (pCMU-4 containing two protospacers targeting *tuf1*, but no homology repair template) and pCM(-cas9) were performed.

As seen in previous experiments with pCMU in *S. coelicolor*, when *cas9* expression was reduced by pCMU-4, exconjugants were observed from all conditions: 11,760 (100%) in the uninduced condition, 8124 (69%) at 0.5 mM, 5712 (49%) at 1 mM and 1440 (12.2%) at 2 mM of theophylline (Table 2, Fig. S4a). pCMU-4tuf gave rise to 10,560 exconjugants (100%) in the uninduced condition, meaning that the basal expression of active Cas9 was most likely not toxic to the cells. Induction with 0.5 mM theophylline seemed to be enough to fully activate the system, as only 36 exconjugants (0.003%) were observed (Table 2, Fig. S4a).

When the same experiments were performed with *S. lividans*, a similar behaviour with pCMU-4 was observed. By using the low-strength promoter, the cells were able to grow at all conditions: 18,000 (100%) in the uninduced condition, 12,516 (88%) at 0.5 mM, 9516 (53%) at 1 mM and 1560 (9%) at 2 mM of theophylline (Table 2, Fig. S4b). When Cas9 activity was tested by using pCMU-4tuf, 20,304 exconjugants (100%) were observed from the uninduced condition, suggesting that basal activity of Cas9 is also low. However, unlike *S. coelicolor*, higher concentrations of theophylline seemed to be needed to fully induce the system, as 240 exconjugants (0.01%) were still observed at 2 mM of theophylline (the highest concentration tested) (Table 2, Fig. S4b). Higher concentration of theophylline was not tested as only 9% of *S. lividans* colonies survived with 2 mM theophylline induction of pCMU-4, indicating that even higher concentration will be too toxic to the cells on solid media.

Conjugation for both *Streptomyces* species tested was successful. The observed differences in efficiency between plasmids pCMU and pCMU-4 depending on the species (*S. coelicolor* versus *S. lividans*), highlight the importance of previously determining the strain tolerance to Cas9 to select an appropriate toolkit before genome editing can be conducted. Without further modifications, the set of experiments and plasmids (pCM2.1, pCM(-cas9), pCMU, pCMUtuf, pCMU-4 and pCMU-4tuf) described in this study could potentially be applied to other actynomycetes in order to test their range of tolerance to Cas9. As *tuf1* is a very conserved gene across species, is very likely that at least one of the two protospacers contained in pCMUtuf and pCMU-4tuf will find its matching sequence in the targeted strain.

### Interruption of glycerol uptake operon using pCMU-4

Once the toxicity of Cas9 levels had been overcome by modifying the promoter strength and a tightly regulated and inducible system was developed for both *S. coelicolor* and *S. lividans* by adjusting the concentration of theophylline, the CRISPR-Cas9 mediated recombination efficiency of this system was tested. For this purpose, the glycerol uptake operon was chosen as a target. This operon comprises four genes: *gylA* (glycerol uptake protein), *gylB* (glycerol kinase) and *gylC* (glycerol-3-phosphate dehydrogenase) are dedicated to the uptake and catabolism of glycerol, while *gylR* encodes a regulatory protein. Inactivation of *gylB* has been shown to be lethal for *S. lividans* when grown on a glycerol-containing medium [35], allowing a screening of CRISPR-Cas9 mediated disruption of the glycerol uptake operon by growth phenotype with and without glycerol. Thus, plasmid pCMU-4dGly was built as described in “Methods” in order to delete a fragment of the chromosome containing 1202 nucleotides of *gylB* coding region and the first 402 nucleotides of the *gylC* coding sequence (Fig. S2). After conjugation, induction on solid media was performed at 0, 0.5, 1 and 2 mM of theophylline. Ten exconjugants from each condition were selected for phenotypic screening by plating out on SMMS with glucose (control) or glycerol as sole carbon source, and for PCR verification with two set of primers, ‘dGy’ and ‘SCO1660′ (Fig. S2, Table S1). Based on previous results with pCMU-4tuf, positive recombinants were expected at 0.5 mM theophylline for *S. coelicolor* and at 2 mM for *S. lividans*. However, all screened colonies were shown to be wild type (data not shown), even at 2 mM for *S. coelicolor*, suggesting that higher concentrations of theophylline might be needed. Therefore, a CRISPR-Cas9 induction in liquid TSB containing 4, 8 and 12 mM theophylline was conducted as described in “Methods” and ten exconjugants from each condition were assessed by both phenotype and PCR.

**Fig. 2:**
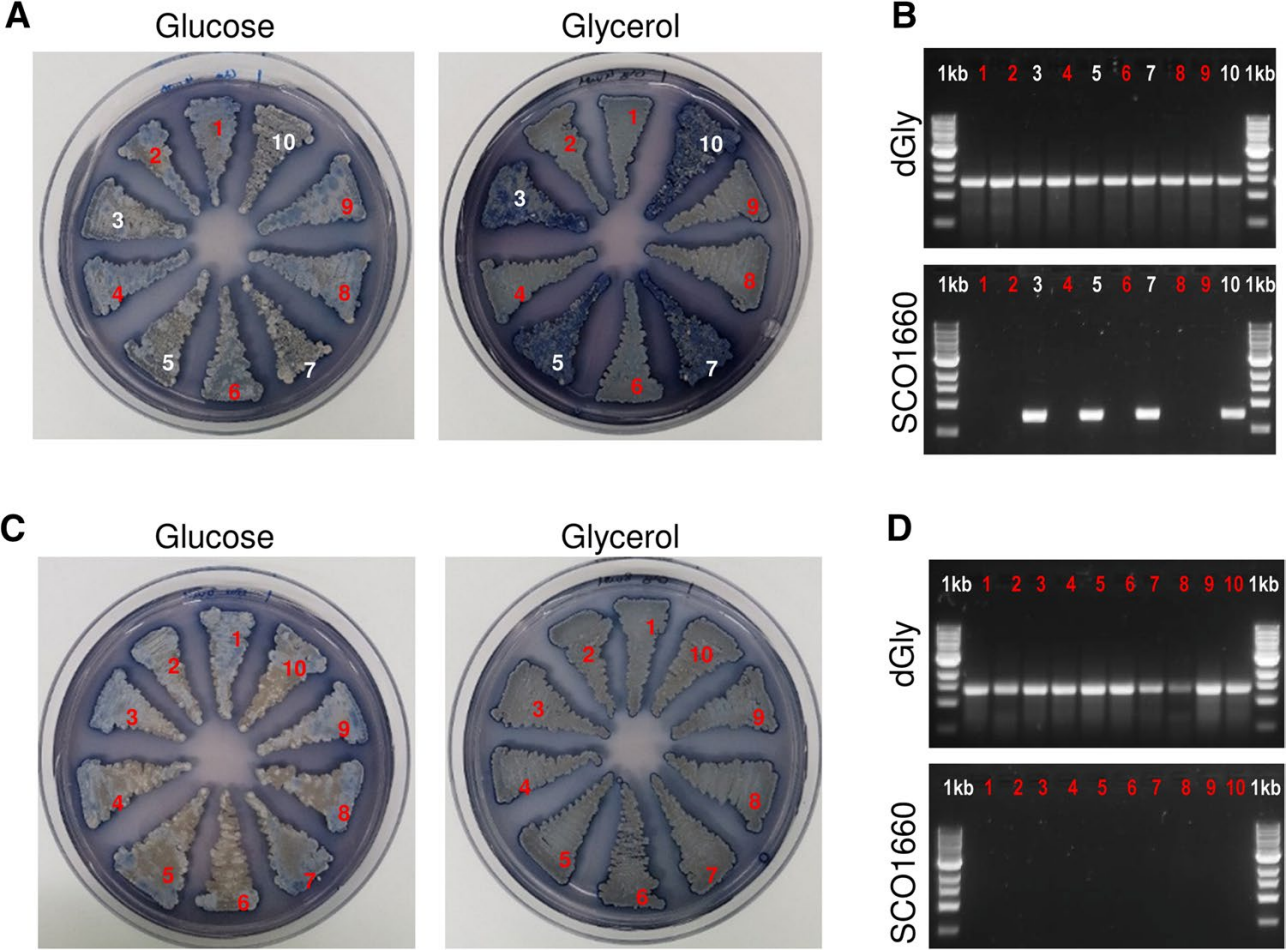
Phenotypic and genotypic screening of *S. coelicolor* colonies after conjugation with pCMU-4dGly and induction with **a** 4 mM and **c** 8 mM of theophylline. PCR verification with set of primers ‘dGly’ and ‘SCO1660 check’ of colonies after induction with **b** 4 mM and **d** 8 mM of theophylline. Positive recombinants are shown in red

For *S. coelicolor*, growth in glycerol was observed for all the ten screened colonies after induction with 4 and 8 mM theophylline, suggesting that the recombination had failed. However, when those colonies were verified by PCR, 6 out of 10 colonies from 4 mM and all 10 out of 10 colonies from 8 mM showed to have the deletions designed in *gylB* and *gylC* (Fig. 2b and d). Though all the exconjugants were able to grow with glycerol, an antibiotic production phenotype was observed between wild-type and positive recombinants, as the former seemed to be producing remarkably more actinorhodin in glycerol. Interestingly, in glucose, despite being less apparent, the situation seemed to be the opposite, with the wild type producing less actinorhodin than the positive exconjugants (Fig. 2a and c).

Unlike *S. lividans, S. coelicolor* possesses two operons dedicated to the glycerol catabolism: the targeted *gylABC* operon (SCO1658-1661) that is glucose repressible and actively uptakes glycerol, and a second less efficient operon that is not under glucose regulation and uses diffusible glycerol [36]. This second operon might be the one allowing *S. coelicolor* mutants to grow in glycerol.

**Fig. 3:**
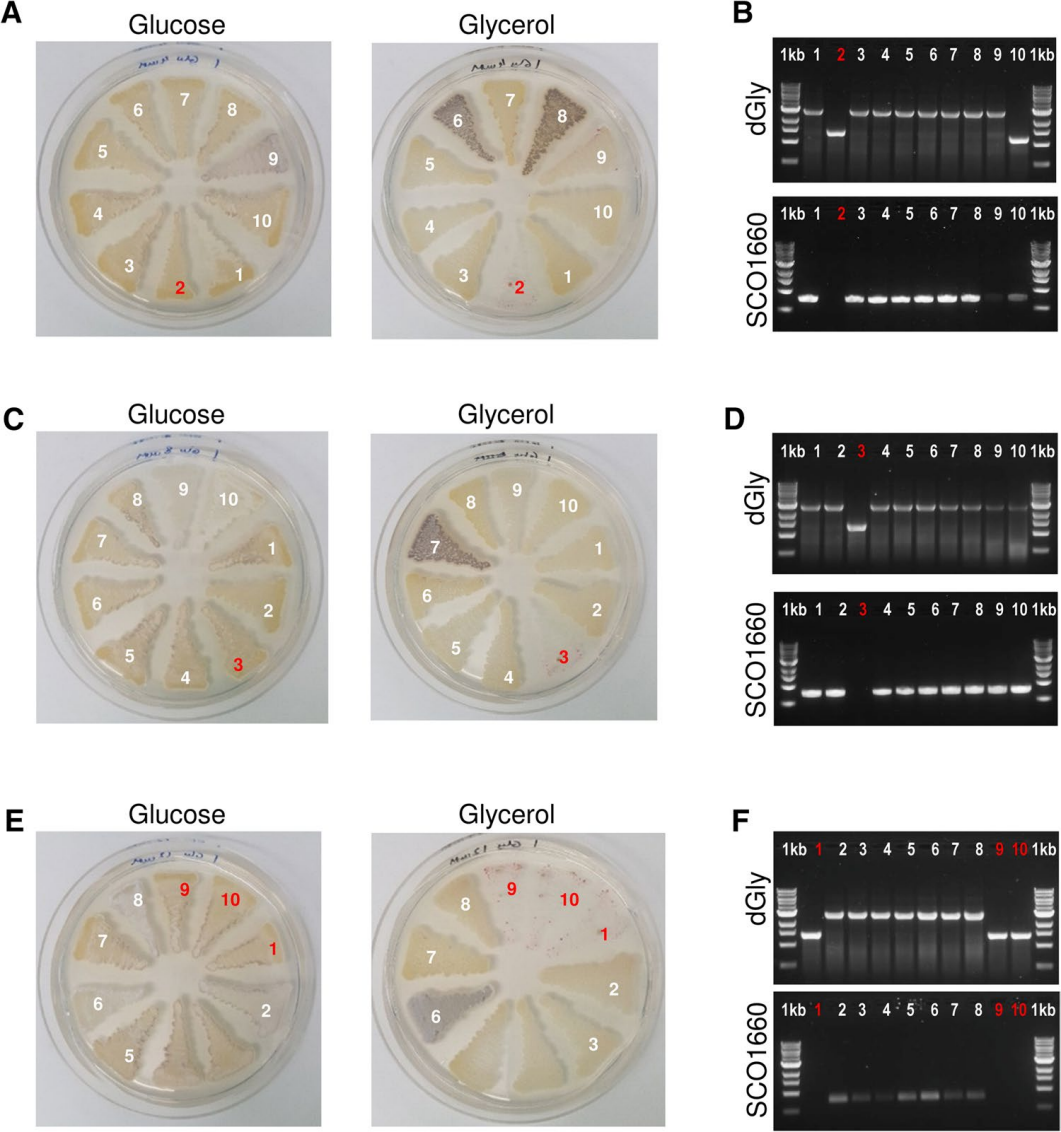
Phenotypic and genotypic screening of *S. lividans* colonies after conjugation with pCMU-4dGly and induction with **a** 4 mM, **c** 8 mM and **e** 12 mM of theophylline. PCR verification with set of primers ‘dGly’ and ‘SCO1660 check’ of colonies after induction with **b** 4 mM, **d** 8 mM and **f** 12 mM of theophylline. Positive recombinants are shown in red

For *S. lividans*, the phenotypic screening revealed those with the correct deletion of the glycerol operon, which are 1 out of 10 recombinants from both 4 mM and 8 mM theophylline induction, and 3 out of 10 recombinants from the 12 mM theophylline induction (Fig. 3a, c and e). In this case, all the positive recombinants showed the expected phenotype where the exconjugants were unable to grow on glycerol after the disruption of the glycerol uptake operon. This was further confirmed by PCR (Fig. 3b, d and f).

The pCMU-4 plasmid constructed in this manuscript has been successfully applied for the disruption of the glycerol uptake operon in both *S. coelicolor* and *S. lividans*. This system has been shown as inducible and dose dependent, as the recombination efficiency increases with higher concentrations of theophylline. The recombination efficiency is also strain-dependent, with 100% of positive recombinants from *S. coelicolor* after induction with 8 mM theophylline versus 10% from *S. lividans* in the same condition. These observations are in agreement with results from pCMU-4/pCMU-4tuf, from which *S. lividans* was predicted to require higher induction compared to *S. coelicolor.*

### pCM4.4, an ermE*p-cas9-driven expression, as an alternative plasmid

While we were able to achieve successful gene editing in both *S. coelicolor* and *S. lividans* using pCMU-4 plasmid, our system still has some drawbacks: (1) As we have an extra step needed for system induction, gene editing occurs in long time frames; (2) Although we were able to use theophylline in sub-lethal concentrations to induce Cas9 expression in both *S. coelicolor* and *S. lividans*, theophylline toxicity might inhibit successful gene editing in other Actinomycetes. To address these potential drawbacks, a new plasmid called pCM4.4 was constructed. As high levels of Cas9 expression from plasmid pCRISPomyces-2 led to Cas9 toxicity in *S. coelicolor*, we replaced Cas9 promoter with a weaker constitutive promoter, ermE*p, to test whether decreasing Cas9 expression could solve Cas9 toxicity issues in this strain. Plasmid pCM4.4 without a gRNA sequence was delivered into *S. coelicolor* by conjugation and we were able to observe thousands of exconjugants after 4 days of incubation at 30 °C. As shown above, we were not able to observe any exconjugants from plasmids pCRISPomyces-2 and pCM2.1, this result suggests the possibility of using constitutive promoters for Cas9 expression, as long as the expression levels are kept on levels which are not toxic to the cells.

**Fig. 4:**
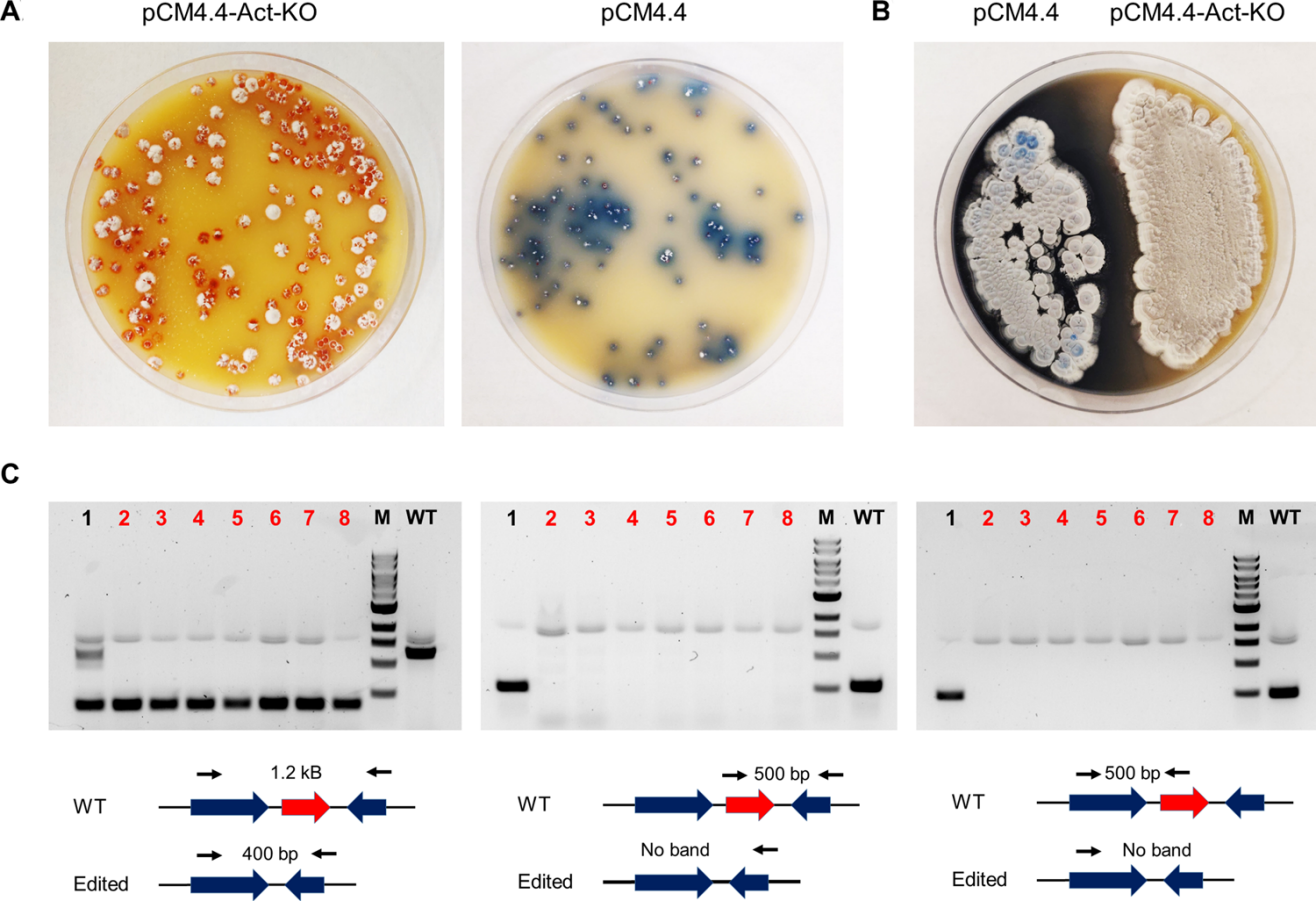
Phenotypic and genotypic screening of *S. coelicolor* exconjugants after conjugation with pCM4.4-Act-KO, pCM4.4. **a** Image of conjugation plates after 5 days of incubation at 30 °C. pCM4.4-Act-KO exconjugants do not show actinorhodin (blue pigment) production. **b** Re-streaked exconjugants for both pCM4.4 and pCM4.4-Act-KO confirm lack of actinorhodin production for edited exconjugants. **c** Genotypic screening of 8 exconjugants confirm deletion of 852 bp target for 7 colonies

We next evaluated pCM4.4 for genome editing in *S. coelicolor* by attempting to knock out an 852-bp DNA sequence corresponding to the actinorhodin biosynthetic gene cluster positive transcriptional regulator *actII*-ORF4 (SCO5085). As titration of this regulator could abolish actinorhodin production in *S. coelicolor* [37], we sought to use actinorhodin production as an indicator for successful genome editing. To perform gene editing, we designed a single gRNA sequence which would lead to Cas9 cleavage in the middle of regulator gene. Two homology arms (~ 1.5 kB in size) on each side of the 852-bp DNA sequence were also provided as template for homologous recombination. The editing plasmid pCM4.4-Act-KO was delivered into *S. coelicolor* by conjugation and an empty pCM4.4 plasmid was also included as control. After conjugation, 100-fold less exconjugants were observed from pCM4.4-Act-KO compared to pCM4.4 which suggests successful Cas9 chromosomal cleavage. As shown in Fig. 4a, none of the exconjugants from pCM4.4-Act-KO showed actinorhodin production (blue pigment) while almost all exconjugants from pCM4.4 showed actinorhodin production. To further confirm this phenotype, single exconjugants from both edited and non-edited cells were streaked on SFM medium (Fig. 4b) which confirmed lack of actinorhodin production in edited cells. Finally, genotyping was performed on eight exconjugants to assess correct gene editing (Fig. 4c). Out of eight exconjugants analysed, seven showed successful deletion of the 852-bp sequence while one exconjugant showed a mixture of edited and non-edited cells resulting in 87.5% editing efficiency.

In summary, we have demonstrated that high levels of Cas9 can be toxic for the cells, translated in a drastically reduced (to even zero) transformation efficiency. Reducing Cas9 levels can remarkably increase transformation efficiency but can also be insufficient for efficient Cas9 activity. Therefore, Cas9 levels should be perfectly balanced within a range that would compromise high transformation efficiency with successful genome editing. Moreover, this range of tolerance is strain dependent and should be experimentally determined. We have developed a set of 5 plasmids (pCM(-cas9), pCMU, pCMUtuf, pCMU-4 and pCMU-4tuf) which have rapidly and easily showed the different range of tolerance to Cas9 expression and activity of *S. coelicolor* and *S. lividans.* Pre-exploration of the range of tolerance with this set of plasmids could be potentially used with any other actinomycete and would be certainly advisable in order to save time and uncertainties when establishing CRISPR-Cas9 genome editing in a new strain. The different range of tolerance to Cas9 activity of *S. coelicolor* and *S. lividans* was further confirmed in the genome editing experiments carried out with plasmid pCMU-4. With this plasmid, Cas9 expression is tightly regulated, allowing optimum transformation efficiency in an initial step with no inductor, followed by successful genome editing by modulation of Cas9 expression with different levels of an inducer. Thus, this plasmid was customised for each strain. The adaptability of this plasmid would be potentially useful for performing CRISPR-Cas9 based genome editing in any other actinomycete, being particularly convenient for those strains especially sensitive to Cas9 expression levels. Alternatively, and for other strains more tolerant to Cas9 levels, we have developed plasmid pCM4.4, in which Cas9 expression has been lowered by using a weaker constitutive promoter. This way, no induction step is necessary, shortening the timeframe of the experiment and avoiding the potential toxicity of theophylline. This work will help to rationally implement CRISPR-Cas9 genome editing to strains in which this technology has been reported as unsuccessful as well as to new actinomycete strains.

## Electronic supplementary material

Below is the link to the electronic supplementary material.

Supplementary file1 (PDF 1389 kb)
