# Supplementary material for: Fine-tuning the regulation of Cas9 expression levels for efficient CRISPR-Cas9 mediated recombination in Streptomyces
Source: J Ind Microbiol Biotechnol. 2020 May 4;47(4):413–23. doi: 10.1007/s10295-020-02277-5 (PMC7244461; doi:10.1007/s10295-020-02277-5)
Supplement: Supplementary file 1 — Supplementary file1 (PDF 1389 kb) [file 10295_2020_2277_MOESM1_ESM.pdf]

Fine tuning the regulation of Cas9 expression levels for efficient CRISPR-Cas9 mediated recombination in *Streptomyces*

Suhui Ye[1,2], Behnam Enghiad[3], Huimin Zhao[3], Eriko Takano[1]

Affiliation:

[1] Manchester Centre for Synthetic Biology of Fine and Speciality Chemicals (SYNBIOCHEM), Manchester Institute of Biotechnology, Department of Chemistry, School of Natural Sciences, Faculty of Science and Engineering, University of Manchester, 131 Princess Street, Manchester M1 7DN, United Kingdom
[2] Present address: Research Group BIONUC (Biotechnology of Nutraceuticals and Bioactive Compounds), Departamento de Biología Funcional, Área de Microbiología, Universidad de Oviedo, Avenida Julián Clavería s/n, 33006, Oviedo, Principality of Asturias, Spain. IUOPA (Instituto Universitario de Oncología del Principado de Asturias), ISPA (Instituto de Investigación Sanitaria del Principado de Asturias).
[3] Department of Chemical and Biomolecular Engineering, and Carl R. Woese Institute for Genomic Biology, University of Illinois at Urbana-Champaign, Urbana, IL 61801, United States

Corresponding author: eriko.takano@manchster.ac.uk

Figure S1. Maps of plasmids used in this study. Annealing sites for primers used for their assembly are indicated.

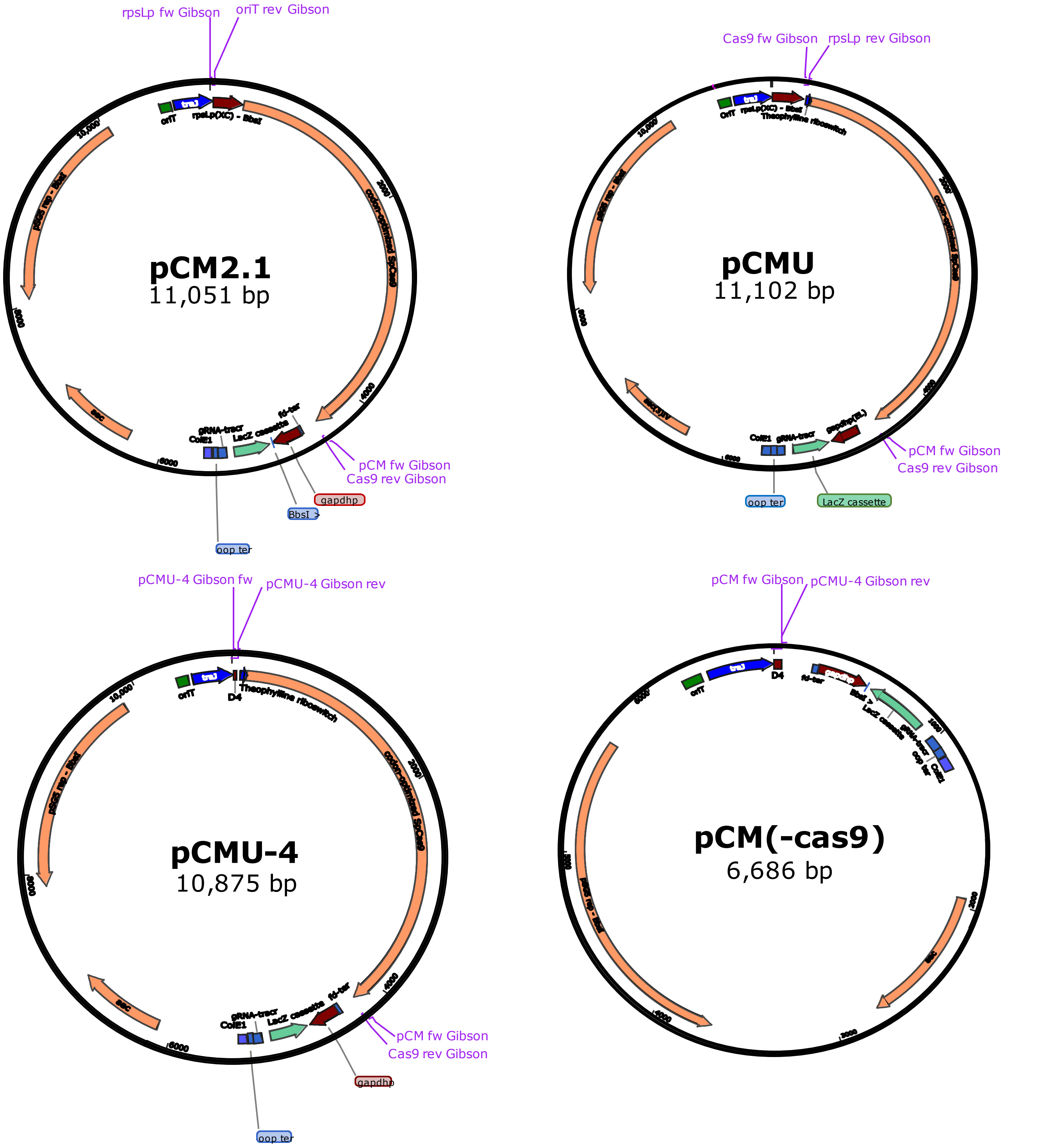

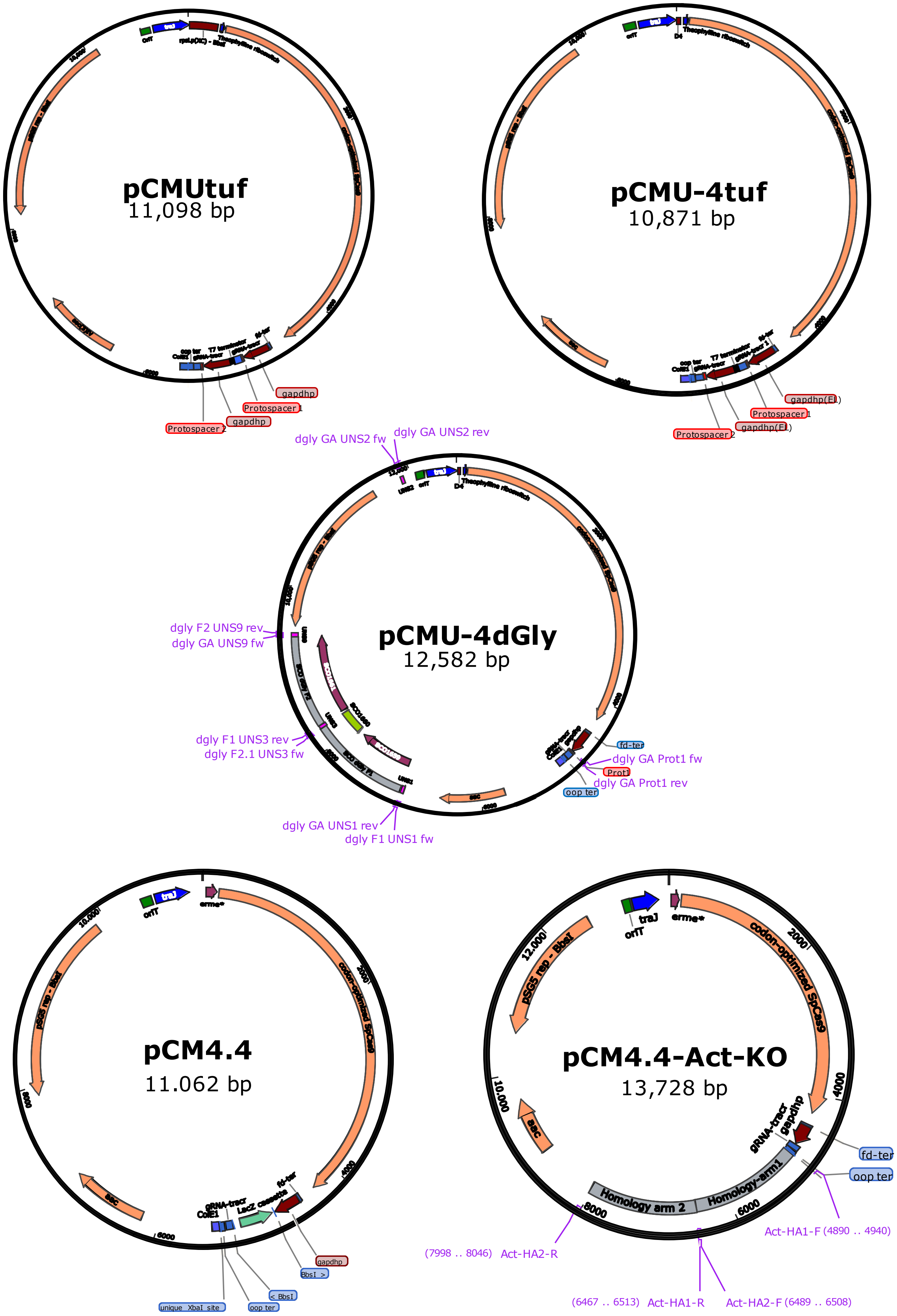
pCMUtuf
11,098 bp
oriT
traJ
Theophylline riboswitch
gapdhp
Protospacer 1
gapdhp
Protospacer 2
pCMU-4tuf
10,871 bp
oriT
traJ
D4
Theophylline riboswitch
gapdhp(F1)
Protospacer 1
gapdhp(F1)
Protospacer 2
dgly GA UNS2 fw
dgly GA UNS2 rev
pCMU-4dGly
12,582 bp
UNS2
oriT
traJ
D4
Theophylline riboswitch
dgly F2 UNS9 rev
dgly GA UNS9 fw
dgly F1 UNS3 rev
dgly F2.1 UNS3 fw
fd-ter
dgly GA Prot1 fw
Prot1
dgly GA Prot1 rev
oop ter
dgly GA UNS1 rev
dgly F1 UNS1 fw
pCM4.4
11.062 bp
oriT
traJ
erme*
codon-optimized SpCas9
pSG5 rep - BbsI
aac
gRNA-tracr
ColE1
LacZ cassette
fd-ter
gapdhp
BbsI >
< BbsI
unique XbaI site
oop ter
pCM4.4-Act-KO
13,728 bp
traJ
oriT
erme*
codon-optimized SpCas9
12,000
pSG5 rep - BbsI
10,000
aac
gRNA-tracr
gapdhp
fd-ter
oop ter
Homology arm 2
Homology-arm1
8000
6000
4000
2000
Act-HA1-F (4890 .. 4940)
(7998 .. 8046) Act-HA2-R
(6467 .. 6513) Act-HA1-R
Act-HA2-F (6489 .. 6508)

Figure S2. Representation of glycerol uptake operon in wild type (WT) and mutant strains, showing annealing sites for primers used to amplify homologous arms for pCMU-4dGly assembly as well as the cutting site for the designed protospacer. Primers used for mutants DNA verification (green and blue) and expected size for the corresponding PCR fragments are shown.

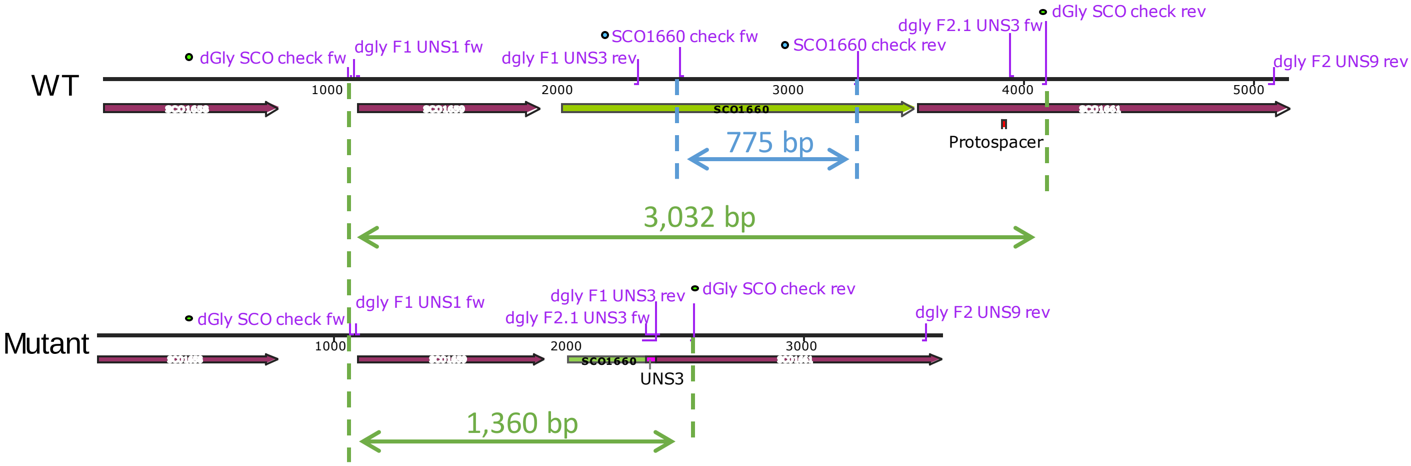

Figure S3. Plates incubated for 6 days against 0, 0.5, 1 and 2 mM theophylline from conjugations with pCM(-cas9) (control of conjugation, lacks *cas9*), pCMU (theophylline riboswitch between *rpsL*p and *cas9*) or pCMUtuf (control of Cas9 activity, pCMU containing 2 protospacers targeting *tuf1* gene but no homology repair template), of A) *S. coelicolor* and B) *S. lividans.* Main features of each plasmid are depicted next to each set of plates for better understanding.

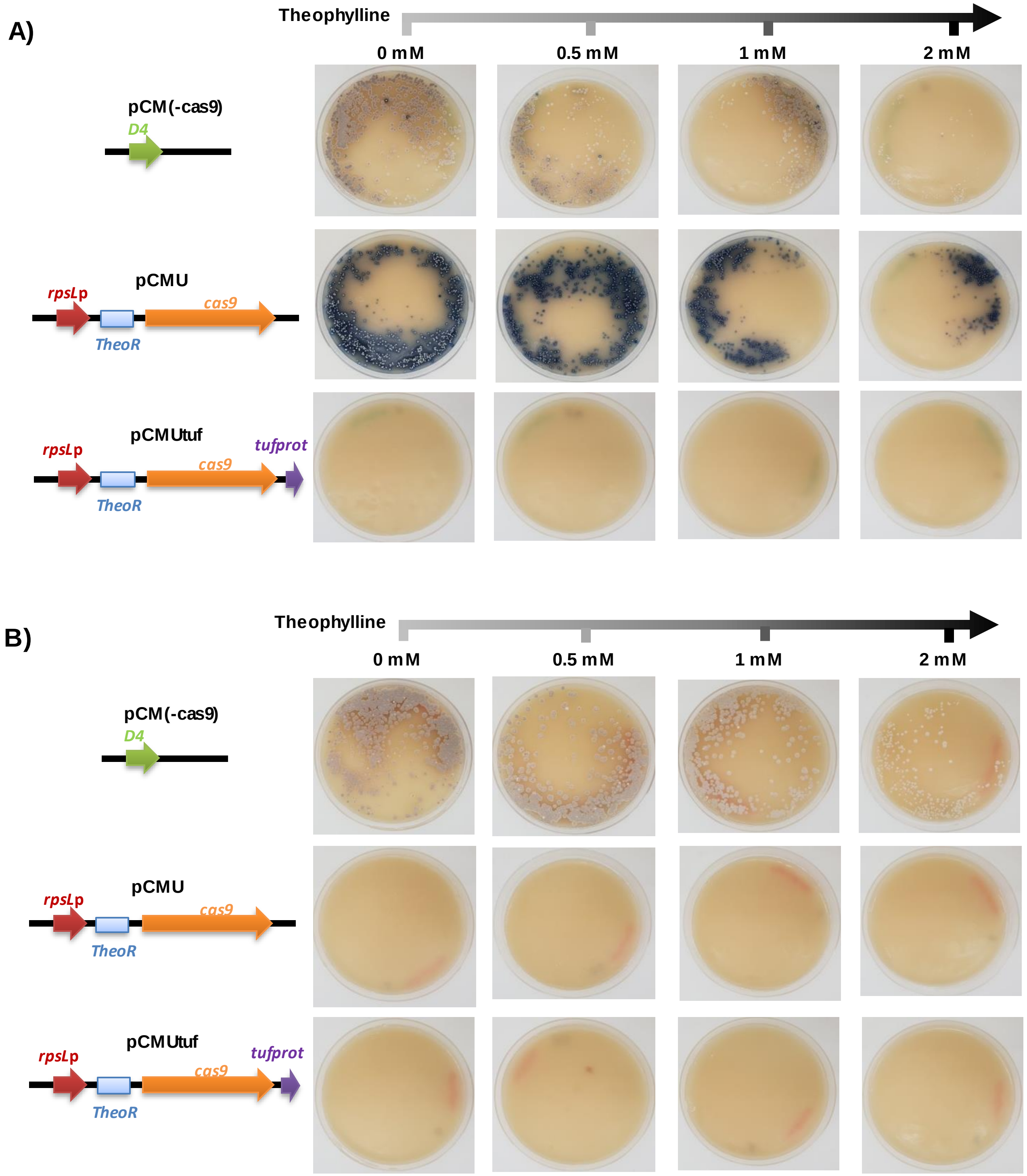

Figure S4. Plates incubated for 6 days against 0, 0.5, 1 and 2 mM theophylline from conjugations with pCM(-cas9) (control of conjugation, lacks *cas9*), pCMU-4 (theophylline riboswitch between *D4* promoter and *cas9*) or pCMUtuf (control of Cas9 activity, pCMU-4 containing 2 protospacers targeting *tuf1* gene but no homology repair template), of A) *S. coelicolor* and B) *S. lividans.* Main features of each plasmid are depicted next to each set of plates for better understanding.

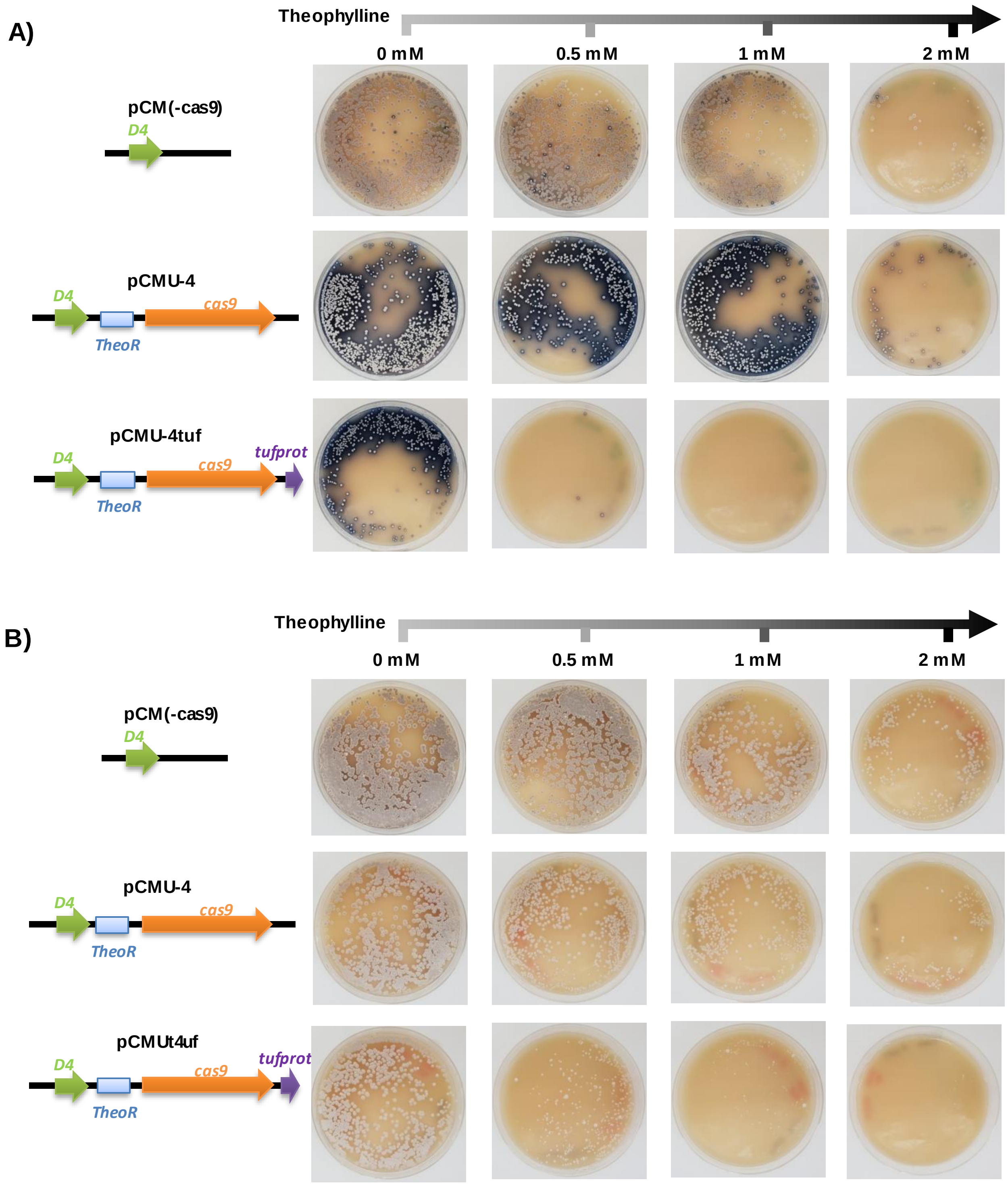

Figure S5. Plates incubated for 10 days against 0, 0.5, 1 and 2 mM theophylline from conjugations with pCM(-cas9) (control of conjugation, lacks *cas9*), pCMU-4 (theophylline riboswitch between *D4* promoter and *cas9*) or pCMUtuf (control of Cas9 activity, pCMU-4 containing 2 protospacers targeting *tuf1* gene but no homology repair template), of *S. lividans.* Main features of each plasmid are depicted next to each set of plates for better understanding.

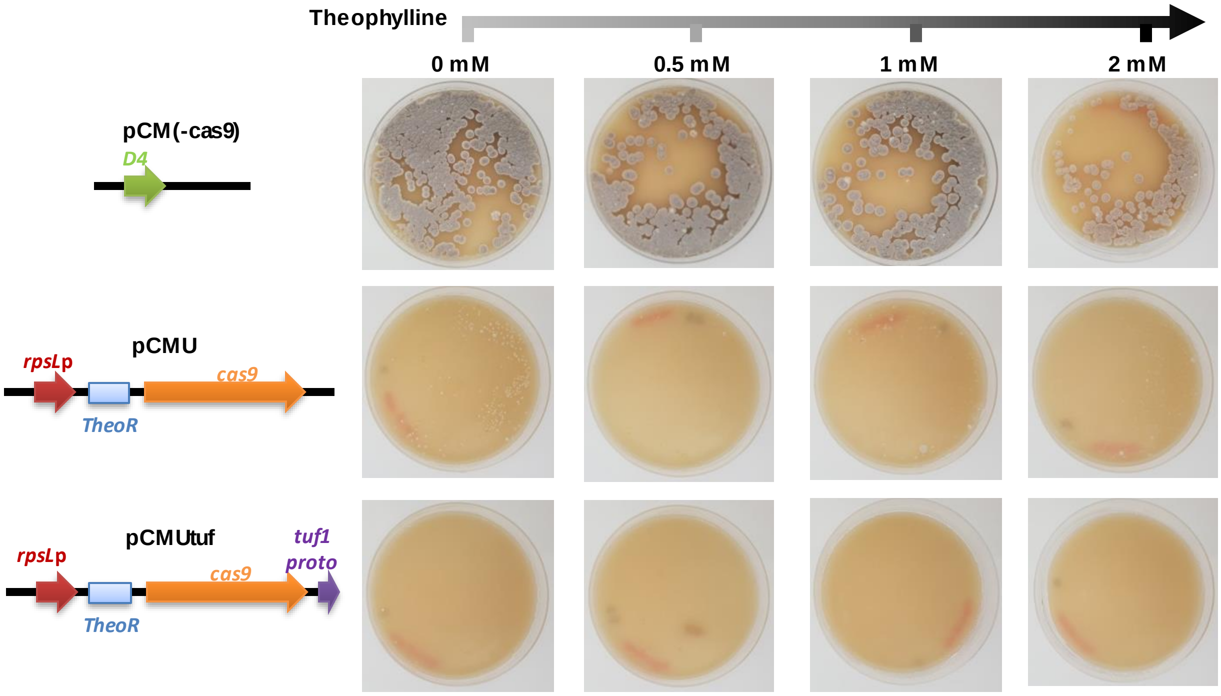

Table S1. Primers and synthetic DNA used in this study

**Primers used for constructs assembly and sequencing**	
**Name**	**Sequence (5' – 3')**
oriT rev Gibson	ctgacttccgcctgcagggctcatggctctgccctcgg
pCM fw Gibson	tgagaattcagatctacgc
rpsLp fw Gibson	gcccgagggcagagccatgagccctgcaggcggaagtc
Cas9 rev Gibson	cagggagtcaaaggccgc
rpsLp rev Gibson	aacctatagtgagtcgtatttacgtctccgtcgtctactcg
Cas9 fw Gibson	gagtagacgacggagacgtaaatacgactcactataggttccggtgatacc
pCMU-4 Gibson fw	cccgtcggctggaaacacttgatccaacttaggatacttccggtaccaatacgactcac
pCMU-4 Gibson rev	ggaagtatcctaagttggatcaagtgtttccagccgacgggtcatggctctgccctcgg
OriT-F	ttgggcagtgcagaaagtgcagaaacctaggtgcaggtcgacggatcttttcc
OriT-R	ccaaaaggagcctttaattgctagctgcaggtccccgggga
dgly GA Prot1 fw	gtatctgaaaggggatacgcgtccgcccttgtacacgggggttttagagctagaaatagc
dgly GA UNS1 rev	gagacgagacgagacagcctgagaatggatgcgagtaatgtcttcttccaaagttgagaa
dgly F1 UNS1 fw	cattactcgcatccattctcaggctgtctcgtctcgtctcgagtcgcgggtgtccagct
dgly F1 UNS3 rev	cgaccttgatgtttccagtgcgattgaggaccttcagtgctgcgggtgtcctgccagac
dgly F2.1 UNS3 fw	gcactgaaggtcctcaatcgcactggaaacatcaaggtcgaagctcggggccggcgtctt
dgly F2 UNS9 rev	cagtgctcttgtgggtccgattcgccagatgataaggaacgtacggcggcgcagcacgtc
dgly GA UNS9 fw	gttccttatcatctggcgaatcggacccacaagagcactgggcagaccctcggctttc
dgly GA UNS2 rev	gcttggattctgcgtttgtttccgtctacgaactcccagcctaggtttctgcactttctgcac
dgly GA UNS2 fw	gctgggagttcgtagacggaaacaaacgcagaatccaagcgtgcaggtcgacggatctt
dgly GA Prot1 rev	ccccgtgtacaagggcggacgcgtatcccctttcagatac
Fragment seq rev	gcgtcgatttttgtgatgct
Act-gRNA-F	acgccgaaagcaatatcgcgcacc
Act-gRNA-R	aaacggtgcgcgatattgctttcg
Act-HA1-F	tcggttgccgccgggcgtttttatctagaatgatcctcacggtcctggtg
Act-HA1-R	ctgcggcgggctggggaactactgatcgagattctccgtctcctggg
Act-HA2-F	tcagtagttccccagcccgc
Act-HA2-R	gcggcctttttacggttcctggcctctagattgccgacggagtccaggc
**Primers used for DNA verification of mutant strains**	
**Name**	**Sequence (5' – 3')**
dGly SCO check fw	ttcggctttccctggacg
dGly SCO check rev	cgtcgttcatctggtcgtcg
SCO1660 check fw	cctgggtcatctggaacct
SCO1660 check rev	atcagcaggttgttggagg
Act-edit-F	aggaatgccagattctattgattcgg
Act-edit-R	acatctgggaggtgtcgacc
Act-WT-F	ttggaatgcagctccgtatcgac
Act-WT-R	tcgtcgatcagggcgaagtag
**Synthetic DNA**	
**Name**	**Sequence (5' – 3')**
tuf1prot	gagacatctttgaagacaaacgcgtcgtgcagcaccttggtaagttttagagctagaaatagcaagttaaaataaggctagtccg<br>ttatcaacttgaaaaagtggcaccgagtcggtgctttttttagcataaccccttggggcctctaaacgggtcttgaggggtttttttggc<br>tgctccttcggtcggacgtgcgtctacgggcaccttaccgcagccgtcggctgtgcgacacggacggatcgggcgaactggccga<br>tgctgggagaagcgcgctgctgtacggcgcgcaccgggtgcggagcccctcggcgagcggtgtgaaacttctgtgaatggcctgt<br>tcggttgcttttttttatacggctgccagataaggcttgcagcatctgggcggctaccgctatgatcggggcgttcctgcaattcttag<br>tgcgagtatctgaaaggggatacgcggtccggccaccctcgcggagtttaagtcttctttcacgtggc
ter-ermE*p-RBS	atgagcacgtccgcgagctggcccgctagcaattaaaggctccttttggagcctttttttacgcggtcgatcttgacggctggcgag<br>aggtgcggggaggatctgaccgacgcggtccacacgtggcaccgcgatgctgttgtgggcacaatcgtgccggttggtaggatcc<br>agcggagcaacggaggtacggacatggacaagaagtacagcatcggcctggacatcggcaccaacagcgtgggctgggcggt<br>catcaccgacgagtacaaggtcccctccaagaagttcaaggtcctgggcaacaccgaccggcactcgatcaagaagaacctgat<br>cggcgccctgctcttcgacagcggcgaaaccgccgaggcgacccgcctgaagcggaccgcccgtcgccgctacacccggcgcaa<br>gaaccgcatctgctacctgcaggagatcttctccaacgagatggcc
